# Boric acid catalysed hydrolysis of peroxyacids†

**DOI:** 10.1039/d3ra01046e

**Published:** 2023-04-14

**Authors:** Michael E. Deary

**Affiliations:** a Faculty of Engineering and Environment, Northumbria University Newcastle upon Tyne NE1 8ST UK michael.deary@northumbria.ac.uk

## Abstract

This study shows for the first time that boric acid catalyses the hydrolysis of peroxyacids, resulting in an approximately 12-fold increase in hydrolysis rate for both peracetic acid (PAA) and 3-chloroperbenzoic acid (MCPBA) when 0.1 M boric acid is present. The maximum rate of hydrolysis occurs at pH 9 and pH 8.4 for PAA and MCPBA respectively. In contrast, carbonate buffer does not enhance the rate of PAA hydrolysis. The reaction was followed by measuring the initial rate of hydrogen peroxide formation using a specific Ti(iv) complexation method. The study of the hydrolysis reaction requires the presence of 2 × 10^−5^ M each of ethylenediaminetetraacetic acid (EDTA) and ethylenediamine tetramethylene phosphonic acid (EDTMP) in all solutions in order to chelate metal ions across the full pH range (3 to 13) that would otherwise contribute to peroxyacid decomposition. Catalysis of peroxyacid hydrolysis is most likely effected by the triganol boric acid acting as a Lewis acid catalyst, associating with the peroxide leaving group in the transition state to reduce the leaving group basicity. The products of the reaction are the well characterised monoperoxoborate species and the parent carboxylic acid. Analysis of the pH and borate dependence data reveals that in addition to a catalytic pathway involving a single boric acid molecule, there is a significant pathway involving either (a) two boric acid molecules or (b) the polyborate species, B_3_O_3_(OH)_4_^−^. Knowledge about catalytic mechanisms for the loss of peroxyacids through hydrolysis is important because they are widely used in reagents in a range of oxidation, bleaching and disinfection applications.

## Abstract 2

Boric acid catalyses the hydrolysis of peroxyacids, with possible pathways involving one and two molecules of boric acid as well as polyborate species.

## Graphical abstract

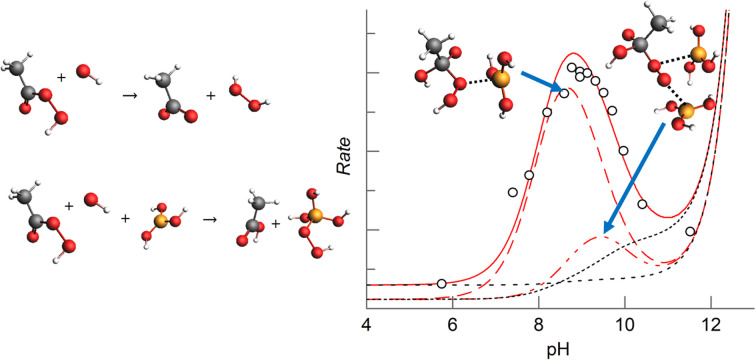

## Introduction

It has previously been shown that boric acid, through the formation of triganol peroxoboric acid at low pH, and monoperoxoborate above pH 8, can catalyse the electrophilic reactions of hydrogen peroxide,^1–3^ the latter most likely proceeding through a mechanism whereby a monocyclic three membered peroxide species, dioxaborirane, is the reactive intermediate.^2,3^ This paper advances our understanding of the catalytic mechanisms of boric acid in aqueous peroxide systems by studying its effect on the rate of peroxyacid hydrolysis, a reaction that yields hydrogen peroxide and the parent carboxylic acid as products. Such knowledge is important because peroxyacids such as peracetic acid are widely used oxidants, for example in laundry detergent formulations,^4^ textile bleaching,^5,6^ wood-pulp bleaching^7^ and in processes where chlorine-free disinfection is required.^8^ The conversion of peroxyacids to the less effective bleaching agent, hydrogen peroxide, is undesirable^9^ and boric acid is often used in bleaching formulations.^4,10^

**Scheme 1:**
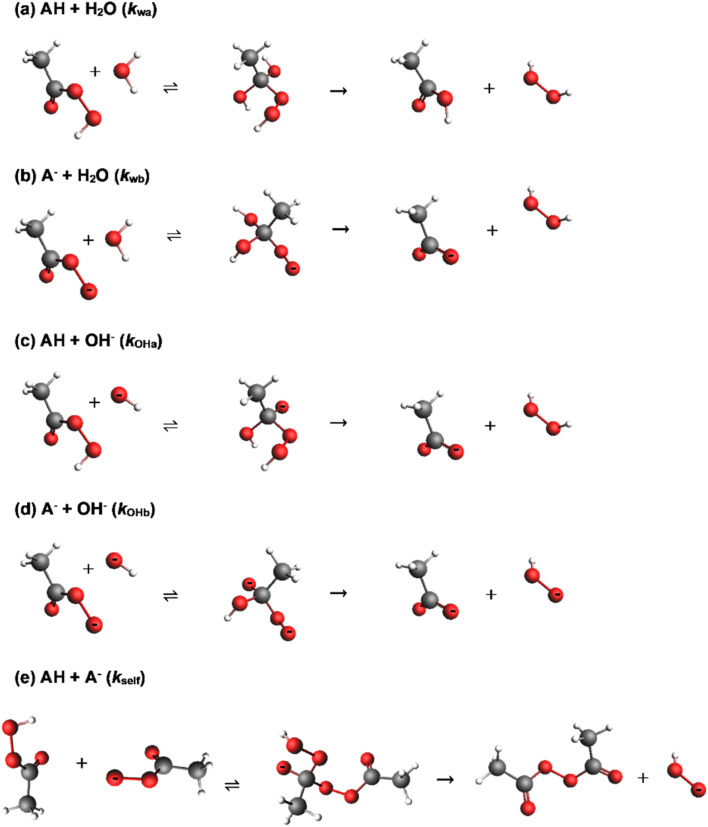
Possible reaction pathways for peracetic acid hydrolysis in mildly acidic to alkaline conditions.^12^ In the sub-headings, AH and A^−^ represent the conjugate acid and conjugate base respectively, and the designated rate constant is that for the overall pathway. The subscript terms ‘w’ and ‘OH’ denote reactions involving H_2_O and OH^−^ respectively and ‘a’ and ‘b’ denote reactions involving the conjugate acid and base of the peroxyacid respectively. Reactions proceed with the attack of the nucleophile on the carbonyl carbon of the peroxyacid, forming a tetrahedral intermediate that decomposes to products in the rate determining step. Note that *k*_wb_ (b) and *k*_OHa_ (c) are kinetically indistinguishable, and the locations of protons in the corresponding tetrahedral intermediate are likely to be interchangeable.

In mildly acidic to alkaline conditions, the uncatalysed hydrolysis of peroxyacids proceeds *via* attack of either water or hydroxide at the carbonyl carbon of either the conjugate acid or conjugate base of the peroxyacid, forming a tetrahedral intermediate that breaks down to the parent acid and hydrogen peroxide in the rate determining step.^11,12^ The reactions have been shown to be first order with respect to peroxyacid and hydroxide ion concentration for perbenzoic acids^11,12^ and for peracetic acid.^13^ This gives four potential hydrolysis pathways, as shown in reactions (a) to (d) of Scheme 1, though reactions (b) and (c) are kinetically indistinguishable. There is also an acid catalysed pathway,^12,14–16^ and a pathway involving two molecules of OH^−^,^11,12^ though these are not likely to be significant under the conditions employed in this work. Yuan *et al.* have reported thermodynamic data that gives Δ*S*^‡^ values of −92.8 and −79.47 J mol^−1^ K^−1^ for the reaction of the hydroxide ion with the conjugate base and with the conjugate acid of peroxyacetic acid respectively (with respective *E*_a_ values of 62.4 and 49.13 kJ mol^−1^). Secco *et al.* have reported Δ*S*^‡^ values of −50.2 J mol^−1^ K^−1^ for the reaction of the peroxybenzoic acid anion with both water and the hydroxide ion (*E*_a_ values of 91.1 and 78.6 kJ mol^−1^), whereas for the hydrolysis pathway involving two hydroxide ions a more negative Δ*S*^‡^ value of −200.8 J mol^−1^ K^−1^ is reported (with an *E*_a_ value of 33.1 kJ mol^−1^).^11^ Secco *et al.* have shown from the hydrolysis of a series of substituted peroxybenzoic acids that all of these reaction paths are sensitive to substituent effects, with Hammett sigma vales of 1.07 for the reaction of the conjugate acid with water, and 1.01 and 1.56 for the reaction between the conjugate base and water or hydroxide respectively.^12^ The hydrolysis reaction is reversible but requires conditions of 15.5 M acetic acid and 0.1 M sulphuric acid catalyst for any appreciable reaction to occur.^17^

In addition to the hydrolysis pathways (a) to (d) in Scheme 1, and of relevance to this study because it is potentially a competing reaction, peroxyacids can decompose *via* self-reaction between the conjugate acid and base forms of the peroxyacid (Scheme 1(e)).^18–24^ Through Edwards' classic ^18^O labelling study, the self-reaction of peracetic acid was found to proceed predominantly (83%) *via* attack of the conjugate base form of peracetic acid at the carbonyl carbon of the conjugate acid, with the remainder of the reaction occurring *via* attack of the conjugate base on the outer peroxidic oxygen of the conjugate acid.^20^ The proportion of the reaction proceeding *via* the latter mechanism increases to 74% for monoperoxyphthalate, where the attack at the carbonyl carbon is sterically hindered.^18^ Electronic effects also appear to be important: *p*-nitroperbenzoic acid, in which the nitro group increases the electrophilicity of the carbonyl carbon, decomposes almost exclusively *via* the former mechanism.^19^ Both routes have oxygen and the parent carboxylic acid as products, though Goodman *et al.* also reported a minor route which yields hydrogen peroxide and diacetyl peroxide: for peracetic acid, this reaction accounted for up to 5% of the total decomposition.^22^

**Fig. 1:**
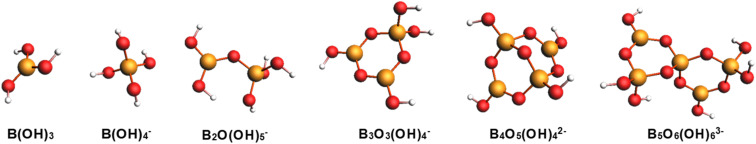
Structures of the main borate species likely to be present under the reaction conditions employed in this study.^27,33^

The study of boric acid catalysis of peroxyacid hydrolysis requires a kinetic model that can account for the complex solution chemistry of boric acid at different concentrations and pHs^2,25–27^ with, in addition to B(OH)_3_ and B(OH)_4_^−^, a range of polyborate species also being present at boron concentrations higher than 0.01 M for solution pHs in the range 6 to 10 (see structures in Fig. 1).^8,27–34^ Evidence from potentiometric,^30,31,35,36^^11^B NMR,^37^ Raman,^27,28,38^ DFT^27,28^ and X-ray absorption studies^39^ indicates the presence of polyborate species containing between two and five boron atoms, with the overall negative charge on the species ranging from zero (B(OH)_3_) to three (B_5_O_6_(OH)_6_^3−^).^32^ Moreover, there is a great deal of inconsistency in the observed formation constants reported from these studies, depending on experimental conditions (particularly pH) and assumptions made as to the prevalent boron species, with reported differences of up to an order of magnitude not uncommon.^35,37^ Ingri's original potentiometric work on boric acid speciation in 0.1 M^36^ and 3 M NaClO_4_ ^31^ proposed that only two polyborate species, B_3_O_3_(OH)_4_^−^ and B_4_O_5_(OH)_4_^2−^ are present in detectable concentrations at boron concentrations less than 0.2 M but that a pentameric polyborate species was required to fit to the data at higher boron concentrations. Mesmer *et al.* found that, in addition, there was a requirement for a dimeric species, B_2_O(OH)_5_^−^.^30^ The nature of the pentameric species is subject to some debate, with (B_5_O_6_(OH)_4_^−^),^36^ (B_5_O_6_(OH)_5_^2−^)^32^ and (B_5_O_6_(OH)_6_^3−^)^32,33^ all being proposed as the most prevalent forms, though the latter is preferred in most of the recent speciation models and studies.^32,33,40^ Nevertheless, at the relatively low boron concentrations used in the current study (<0.2 M), the pentameric form will not be a significant species.

Because of the complexity of boric acid speciation, recent studies have used aqueous chemistry speciation models based on thermodynamic databases to calculate species distributions under different conditions of pH, ionic strength and temperature.^27,32,33^ The models allow activity coefficients and specific ion interactions to be accounted for in calculating equilibrium concentrations.^33^ The mixed-solvent electrolyte (MSE) model of Wang *et al.*^33^ developed for OLI Studio (OLI Systems, Inc), and which is based on the equilibrium studies of Mesmer *et al.*^30^ and Palmer *et al.*^40^ was used in the current study to calculate boron species concentrations for a range of boron concentrations and pHs. The species equilibria used in this model are shown in Table 1.

**Table 1:** Main equilibria for boron species used in the current study^33^

Equilibrium
B(OH)_3_ + H_2_O ⇌ B(OH)_4_^−^ + H^+^
2B(OH)_3_ ⇌ B_2_O(OH)_5_^−^ + H^+^
3B(OH)_3_ ⇌ B_3_O_3_(OH)_4_^−^ + 2H_2_O + H^+^
4B(OH)_3_ ⇌ B_4_O_5_(OH)_4_^2−^ + 3H_2_O + 2H^+^
5B(OH)_3_ ⇌ B_5_O_6_(OH)_6_^3−^ + 3H_2_O + 3H^+^

The purpose of this study is to investigate the effect of boric acid on the hydrolysis of peracetic acid (PAA) and 3-chloroperbenzoic acid (MCPBA), thus expanding our understanding of the catalytic behaviour of this molecule. Moreover, the study aims to provide information on peroxyacid hydrolysis that will be useful to those processes and industries where these compounds are used. The study was carried out by measuring hydrolysis rates in the presence and absence of boric acid over a pH range of 3 to 13.

## Experimental

### Peroxyacid hydrolysis

All reagents were obtained from Sigma-Aldrich Ltd, and were of analytical grade, with the exception of Titanium(iv) sulfate (15% w/v technical grade, Fisher Scientific). A stock solution of MCPBA (*ca.* 7.5 mM) was prepared as previously described.^41^ To prepare the PAA stock solution (*ca.* 0.4 M), it was first necessary to remove the hydrogen peroxide that is present in reagent PAA solutions by using the method of Davies and Deary.^4^

Peroxyacid hydrolysis was followed by measuring hydrogen peroxide formation using a specific spectrophotometric Ti(iv) complexation method, as previously described.^1^ Observed first order rate constants for hydrolysis (*k*_obs_ and *k*_obsB_ in the absence and presence of boric acid respectively) were obtained from the quotient of the initial rate and the initial peroxyacid concentration. To study the uncatalysed hydrolysis of peroxyacid, solutions of peroxyacid were adjusted to the required pH using 0.5 M NaOH,^11,12^ with the ionic strength maintained at 0.2 M using NaNO_3_. To study of the effect of boric acid on the hydrolysis of peroxyacids, boric acid (0.5 M), NaOH (0.5 M), NaNO_3_ (2 M), distilled water and peroxyacid were mixed together at various ratios to achieve a range of pHs and desired boric acid concentrations at an ionic strength of 0.2 M. For reactions in the presence and absence of boric acid, initial concentrations of PAA and MCPBA were *ca.* 4.0 mM and 3.5 mM respectively. The pH was measured in the reaction solution, both at the beginning and end of the reaction monitoring period.

It is important to note the necessity of adding 2 × 10^−5^ M each of ethylenediaminetetraacetic acid (EDTA) and ethylenediamine tetramethylene phosphonic acid (EDTMP) to all of the reaction solutions, at least 1 hour before use, so as to chelate metal ions across the full pH range used (3 to 13) that would otherwise catalyse peroxide decomposition. Initial studies conducted using either EDTA or EDTMP alone, showed an increase in the decomposition of total peroxide over the course of the runs (typically 2 hours). When EDTA and EDTMP are both present, competing metal ion catalysed side reactions are minimised, allowing approximately 90% conversion to hydrogen peroxide at 0.1 M borate at pH 10.2. The sensitivity of these systems to metal ions, and the importance of using chelating agents other than EDTA, has previously been reported in the literature.^13,21^ It is also notable that the hydrogen peroxide formed during peracetic acid hydrolysis in the presence of boric acid shows remarkable stability: a 3.4 mM solution of hydrogen peroxide with 0.1 M boric acid at pH 9.3 was found to retain over 90% of its activity after eight months. This can be ascribed to the formation of peroxoborate species^3^ that confer stability with respect to peroxide decomposition reactions.

### Calculation of equilibrium concentrations of boron species in hydrolysis reaction solutions

For hydrolysis reactions carried out in the presence of boric acid, concentrations of B(OH)_3_, B(OH)_4_^−^, B_2_O(OH)_5_^−^, B_3_O_3_(OH)_4_^−^, B_4_O_5_(OH)_4_^2−^ and B_5_O_6_(OH)_6_^3−^ in the reaction solution were calculated using OLI studio version 9.6 (OLI Systems Inc) for each of the experimental conditions used in this study. OLI Studio's mixed-solvent electrolyte (MSE) model was selected as the thermodynamic database, as this incorporates a recent thermodynamic dataset for boron species developed by Wang *et al.*^33^ based on the equilibria, activity coefficients and ion interaction terms determined by Mesmer *et al.*^30^ as modified by Palmer *et al.*^40^ OLI Studio has been widely used in the literature to study aqueous chemistry speciation,^42^ including for the quantitative analysis of Raman spectroscopic data on polyborate speciation in aqueous solutions of boric acid.^27^ This approach presents an advantage over the use of published formation constants that were often determined under conditions very different to those used in the present study, *e.g.* 3 M NaClO_4_.^31^ The use of the OLI Studio model allows the effect of ionic strength and the presence of NaNO_3_ to be taken into account when calculating equilibrium concentrations, as well as enabling species distributions to be calculated over a range of temperatures.

The inputs into OLI Studio were: solution pH, temperature (25 °C), boric acid concentration, NaNO_3_ concentration (used to maintain the ionic strength at 0.2 M) and the identity of the titrant used to reach the solution pH (NaOH). The ‘single point’ calculation was used to determine the equilibrium concentrations that were subsequently exported to the data analysis software (Grafit version 7.0.3 ^43^). In addition, for the pH dependence study, equilibrium concentrations of the boron species were determined for pHs in the range 4 to 13 at intervals of 0.01 pH units using the ‘survey’ calculation. The pH-dependent speciation calculation was repeated at temperatures of 50 °C and 75 °C, in addition to that at 25 °C.

### Curve-fitting analysis

Best-fit values of rate constants for various equations describing the dependence of (a) *k*_obs_ as a function of pH and (b) *k*_obsB_ as a function of both pH and the concentration of boron species, were obtained from non-linear least squares analysis using Grafit version 7.0.3 with proportional weighting.^43^ Concentrations of boric acid and polyborate species for a specific pH and boric acid concentration were obtained as described in the previous section. For PAA, best fits were obtained by simultaneously fitting to both pH and borate dependence data, thus ensuring a more robust determination of the respective rate constants. For MCPBA, fits were made only to pH dependence data.

## Results and discussion

### Peroxyacid hydrolysis in the absence of boric acid

**Fig. 2:**
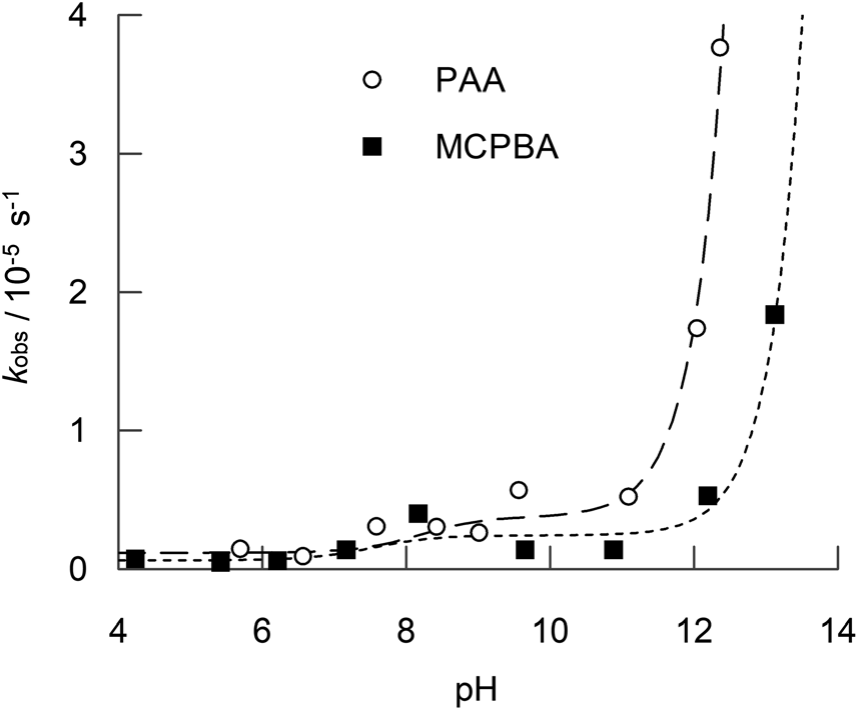
pH dependence for the hydrolysis (*k*_obs_) of PAA and MCPBA at 25 °C. No boric acid was present. Initial peroxyacid concentrations, [P]_t_, were 4.0 mM and 3.5 mM for PAA and MCPBA respectively. Ionic strength was maintained at 0.2 M with sodium nitrate. All solutions contained 20 μM each of ETDA and EDTMP. The curves are the best fits to eqn (1) for the rate constants listed in Table 2. The corresponding data is included in Table S1 in the ESI.†

A pH dependence for the hydrolysis of PAA and MCPBA in the absence of boric acid is shown in Fig. 2. The curves are best fits to eqn (1), where *k*_obs_ is the observed first order rate constant for hydrolysis; [P]_t_ is the total peroxyacid concentration; and *k*_wa,_*k*_wb_ and *k*_OHb_ are hydrolysis pathways, as defined in Scheme 1. [AH] and [A^−^] are the concentrations of the conjugate acid and conjugate base of the peroxyacid respectively, which were determined from eqn (2) and (3), where *K*_p_ is the peroxyacid dissociation constant (6.31 × 10^−9^ M and 2.95 × 10^−8^ M for PAA^44^ and MCPBA^45^ respectively).

Although it is listed in Scheme 1, *k*_OHa_ was omitted from eqn (1) because it is kinetically indistinguishable from *k*_wb_. Secco *et al.* used *k*_wb_ in their analysis of the hydrolysis of substituted perbenzoic acids^12^ and so its use in the current analysis allows a direct comparison of rate constants. *k*_self_ was also omitted from eqn (1), as preliminary analysis indicated that it does not improve the overall fit. Moreover, this pathway, which produces H_2_O_2_ as a product, accounts for only about 5% of the total peroxyacid decomposition that occurs as a result of the overall self-reaction.^22^

1 *k*_obs_ = (*k*_wa_ [AH] + *k*_wb_ [A^−^] + *k*_OHb_ [OH] [A^−^])/[P]_t_

2 [AH] = [P]_t_ [H^+^]/(*K*_p_ + [H^+^])

3 [A^−^] = [P]_t_ − [AH]

Whilst the analysis shown in Fig. 2 gives a good fit to the data for both PAA and MCPBA, yielding the rate constants shown in Table 2, there are is some scatter in the data that is indicative of the sensitivity of this reaction to impurities. The determined rate constants are consistent with those obtained by Secco *et al.*^12^ for a range of substituted perbenzoic acids: 1.7 to 18.8 × 10^−7^ s^−1^ for *k*_wa_, 3.0 to 28.0 × 10^−6^ s^−1^ for *k*_wb_, and 1.14 to 48.0 × 10^−4^ dm^3^ mol^−1^ s^−1^ for *k*_OHb_. Yaun *et al.* determined a value of *k*_OHb_ = 93.4 × 10^−4^ dm^3^ mol^−1^ s^−1^ for PAA at 40 °C,^13^ which is ten-fold higher than the value at 25 °C reported in this study. The same authors also quoted a value of *k*_OHa_ = 7.38 dm^3^ mol^−1^ s^−1^ for PAA at 40 °C.^13^*k*_OHa_ is kinetically equivalent to *k*_wb_ used in this work; however, when *k*_OHa_ is used instead of *k*_wb_ for curve fitting in the present study at 25 °C, values of 1.41 ± 0.28 dm^3^ mol^−1^ s^−1^ and 4.25 ± 1.01 dm^3^ mol^−1^ s^−1^ are obtained for PAA and MCPBA respectively.

**Table 2:** Rate constants for the hydrolysis of PAA and MCPBA in the absence of boric acid, obtained from the best fit to eqn (1) as detailed in the text

Rate	PAA	MCPBA
*k*_wa_/10^−7^ s^−1^	11.69 ± 7.05	6.27 ± 5.89
*k*_wb_/10^−6^ s^−1^	3.76 ± 0.76	2.43 ± 0.58
*k*_OHb_/10^−4^ dm^3^ mol^−1^ s^−1^	8.13 ± 0.38	0.69 ± 0.05

### Boric acid speciation

**Fig. 3:**
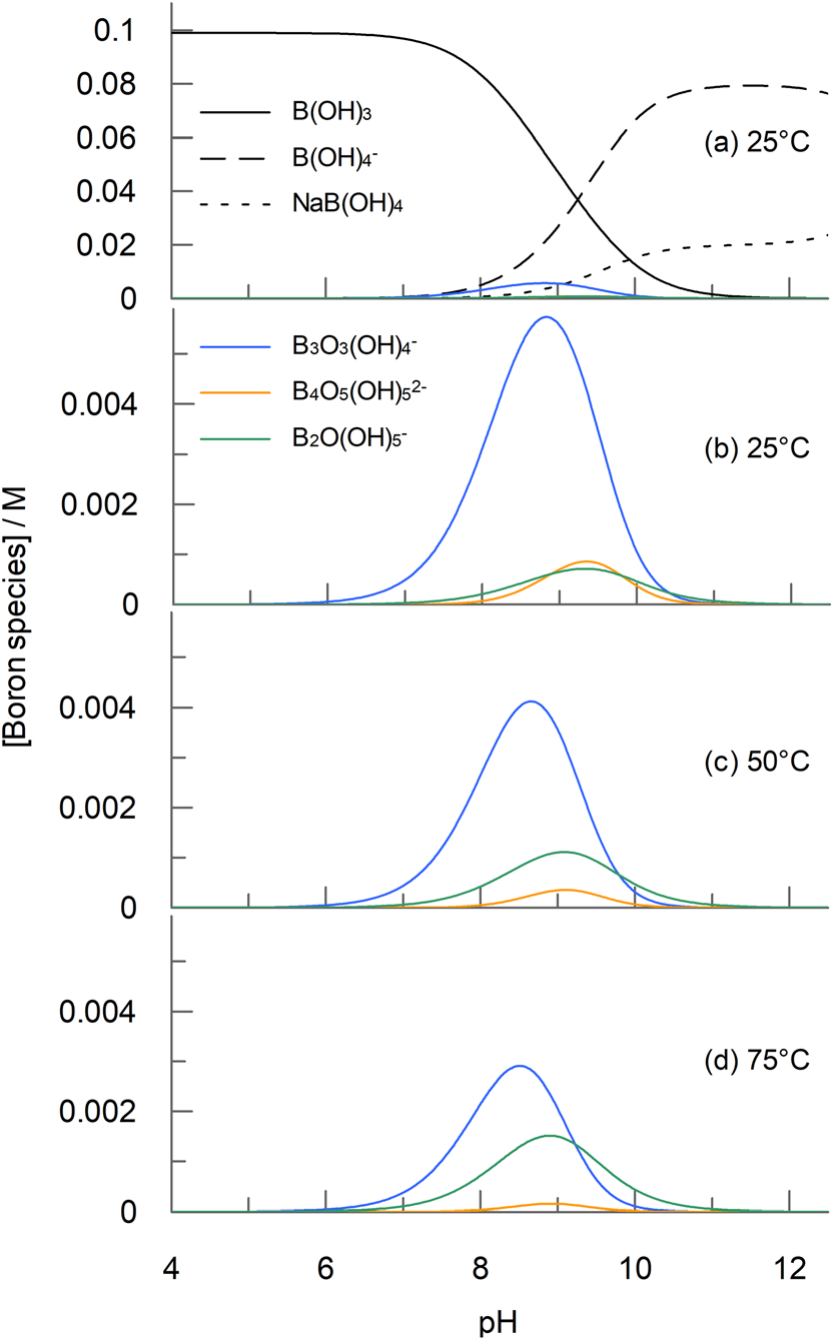
Distribution of significant boron species as a function of pH for a 0.1 M boric acid solution. Panel (a) shows the profile for all species at 25 °C and panels (b) to (d) show only the polyborate distribution for three different temperatures. Concentrations were calculated using OLI Studio 9.6, as described in the text. The concentration of B_5_O_6_(OH)_6_^3−^ is negligeable.

Before discussing the effect of boric acid on peroxyacid hydrolysis, it is useful to first understand the distribution of boron species as a function of pH, as calculated by OLI Studio 9.6 using the MSE model for the boric acid system developed by Wang *et al.*^33^Fig. 3 shows pH profiles for concentrations of the boron species identified in Fig. 1 (and Table 1), for a 0.1 M solution of boric acid. The effect of temperature on species distribution is also shown. Fig. 3(a) shows that B(OH)_3_ and B(OH)_4_^−^ are the predominant species, depending on pH (with the sodium complex of B(OH)_4_^−^ also present at higher pH). Of the polyborate species, B_3_O_3_(OH)_4_^−^ is the most prevalent, though at 25 °C it represents a maximum of only 5% of the total boron species. B_2_O(OH)_5_^−^ and B_4_O_5_(OH)_4_^2−^ are also predicted to be present in smaller amounts, though the concentration of B_5_O_6_(OH)_6_^3−^ is negligeable. Fig. 3(b)–(d) show that the concentration of B_3_O_3_(OH)_4_^−^ is predicted to decrease with increasing temperature, whilst the concentration of B_2_O(OH)_5_^−^ increases.

### Peroxyacid hydrolysis in the presence of boric acid

**Fig. 4:**
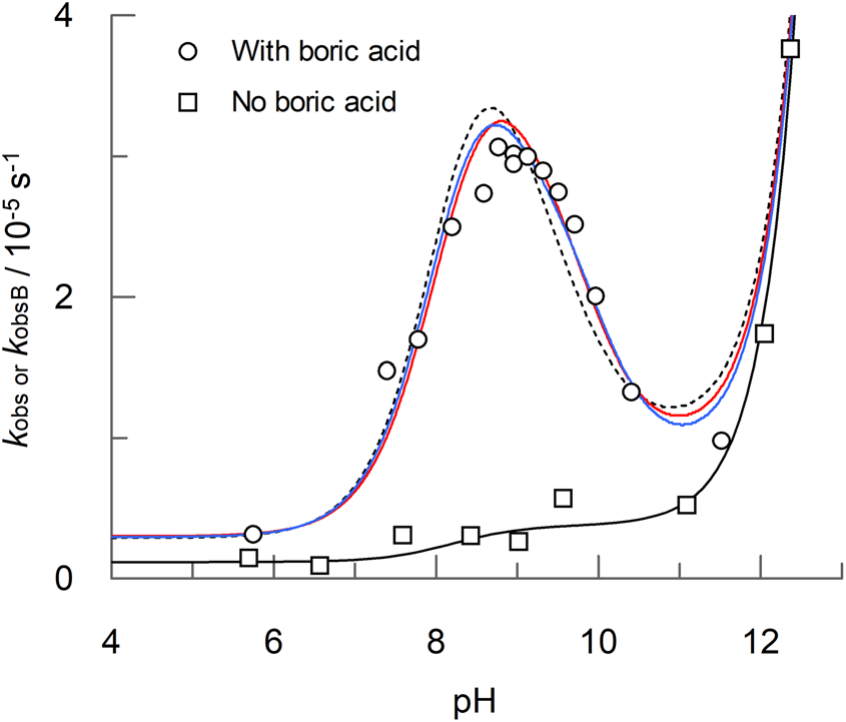
pH dependence of PAA hydrolysis in the presence (*k*_obsB_, open circles) and absence (*k*_obs_, open squares) of 0.1 M boric acid at 25 °C. The initial PAA concentration, [P]_t_, was 4.0 mM. Ionic strength was maintained at 0.2 M with sodium nitrate. All solutions contained 20 μM each of ETDA and EDTMP. The solid black curve is the best fit to eqn (1) for the reaction carried out in the absence of boric acid. The red and blue solid curves are the best fits to eqn (4) and (5) respectively for reactions carried out in the presence of boric acid, with the rate constants listed in Tables 3 and 4 respectively. The dotted curve is the best fit to either eqn (4) or (5) when *k*_OHb(BB)_ or *k*_OHb(B3)_, respectively, are set at zero. The corresponding data is included in Table S2 in the ESI.†

**Fig. 5:**
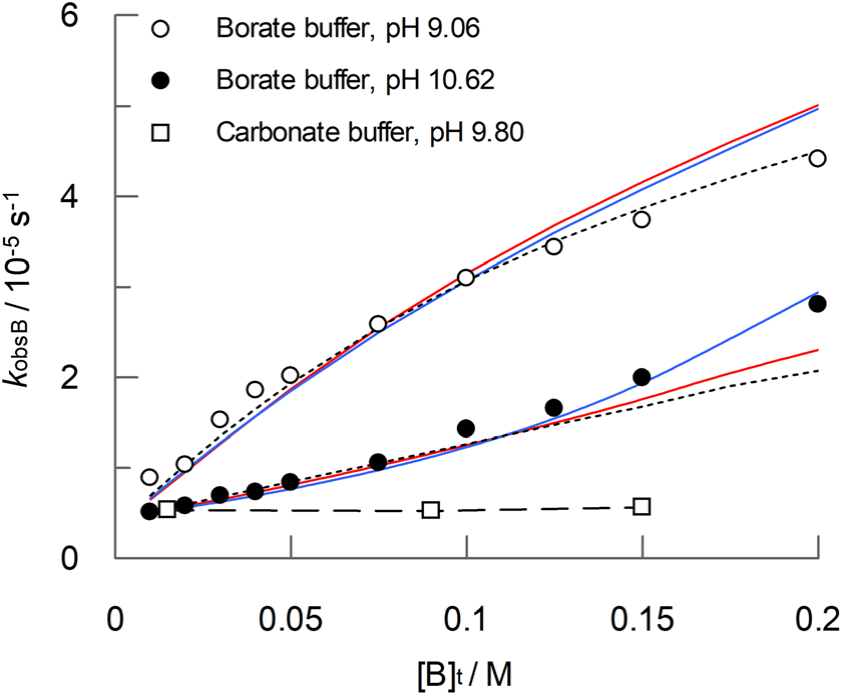
Dependence of *k*_obsB_ on boric acid concentration ([B]_t_) at pH 9.06 and pH 10.62. Also shown for comparison is a concentration dependence of PAA hydrolysis for carbonate buffer at pH 9.8 (the corresponding dashed curve is a guide for the eye). Reaction conditions are as stated in Fig. 4. The red and blue solid curves are the best fits to eqn (4) and (5) respectively for reactions carried out in the presence of boric acid, with the rate constants listed in Tables 3 and 4 respectively. The dotted curve is the best fit to either eqn (4) or (5) when *k*_OHb(BB)_ or *k*_OHb(B3)_, respectively, are set at zero. The corresponding data is included in Table S3 in the ESI.†

In the presence of 0.1 M boric acid, Fig. 4 shows that for PAA there is a clear accelerating effect on the observed rate of hydrolysis, *k*_obsB_, across a range of pHs, with a maximum at pH 9.0, for which the rate is 12-fold higher than in the absence of boric acid. The accelerating effect of boric acid on PAA is further demonstrated by the concentration dependence at two different pHs, shown in Fig. 5. The curves for the boric acid catalysed reactions shown in Fig. 4 and 5 are best fits to equations for proposed reaction schemes that are discussed later. In contrast to boric acid, carbonate buffer has no effect on the rate of formation of hydrogen peroxide, as shown in the comparison concentration dependence shown in Fig. 5.

In modelling the hydrolysis of peroxyacids in the presence of boric acid, it can be speculated that reactions (a) to (d) in Scheme 1 may be catalysed by a molecule of B(OH)_3._ Nevertheless, because *k*_wb_ and *k*_OHa_, are kinetically equivalent, only one of these terms can be included in the analysis, and the decision was to use *k*_wb_ for reasons already stated that relate to the availability of comparable hydrolysis data for the uncatalysed reaction. Also considered was the involvement of two boric acid molecules and of the polyborate species, B_3_O_3_(OH)_4_^−^ which has a trigonal boron atom and could participate in similar catalytic mechanisms to B(OH)_3_. The involvement of the other polyborate species, was not considered because at 25 °C, our speciation modelling shows that B_3_O_3_(OH)_4_^−^ is by far the dominant of these species. The involvement of the tetrahedral borate anion, B(OH)_4_^−^, *via* a catalytic mechanism facilitating proton transfer reactions during the breakdown of the tetrahedral intermediate was considered highly unlikely because the p*K*_a_ of the first dissociation step for one of these hydroxyl groups is *ca.* 22.^46^

**Fig. 6:**
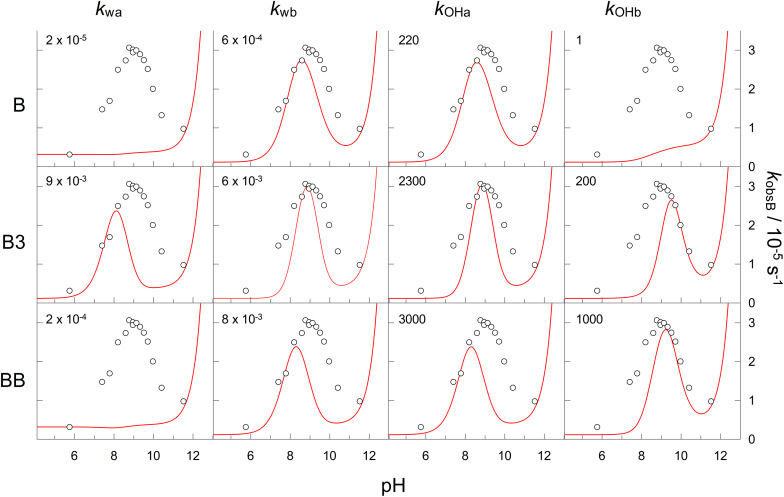
Simulated pH profiles (red curves) for the hydrolysis pathways shown in Scheme 1, catalysed by B(OH)_3_, B_3_O_3_(OH)_4_^−^ and 2B(OH)_3_, denoted B, B3 and BB respectively. The corresponding rate constants are shown on the plots. The open circles are the experimentally determined *k*_obsB_ values for PAA hydrolysis in the presence of 0.1 M boric acid at 25 °C with *I* = 0.2 M.

Even with a restriction on the number of potential catalytic boron species that are considered (B(OH)_3_, B_3_O_3_(OH)_4_^−^ and 2B(OH)_3_), the curve fitting analysis is complex, and is sensitive to the initial estimates provided for the rates. Therefore, initial estimates of rates used in the curve fitting were obtained from a simulation of the pH dependence of *k*_obsB_, as shown in Fig. 6, based on OLI Studio determined boron species concentrations and chosen reaction rates. The simulation was carried out using Grafit Version 7.^43^ Rate constants used in the simulation, for each combination of hydrolysis pathway (Scheme 1) and boron catalyst, were chosen so the they approximately matched the experimental data for PAA hydrolysis in the presence of boric acid (open circles in Fig. 6). The values for the rate constants are shown in each panel of Fig. 6 and they were subsequently used as an upper limit for the initial estimate in the curve fitting.

In addition, the simulation shown in Fig. 6 provides several important pieces of information about the boric acid catalysed hydrolysis of PAA. Firstly, that no single combination of hydrolysis pathway and catalytic species can explain the experimentally determined results; therefore, there must be two or more catalytic pathways. Secondly, that there are several catalytic pathways that when combined should be able to explain the experimental pH profile of PAA hydrolysis. Finally, that several of the catalytic pathways produce similar pH profiles. This latter point is important and requires that pH and boric acid concentration data be fitted simultaneously in order to elucidate the most likely catalytic pathways from those presented in Fig. 6. Therefore, the non-linear least squares curve-fitting analysis was carried out for the terms involving boron species (and including the uncatalysed hydrolysis term) simultaneously for both the pH (Fig. 4) and boric acid dependence (Fig. 5) data.

Two sets of reaction pathways, described by eqn (4) and (5), were found to explain the experimental data with a similar goodness of fit. Both equations incorporate the previously defined terms for the uncatalysed reaction (*k*_obs_, eqn (1)), and both include terms for the reaction, catalysed by a single B(OH)_3_ molecule, of (a) the conjugate acid of PAA with a water molecule (*k*_wa(B)_); (b) the conjugate base of PAA with a water molecule (*k*_wb(B)_) and (c) the conjugate base of PAA with a hydroxide ion (*k*_OHb(B)_). However, eqn (4) and (5) differ with respect to the fourth catalytic term which involves the reaction of the conjugate base of PAA with a hydroxide ion. In eqn (4), the catalyst is two molecules of B(OH)_3_, whereas in eqn (5) the catalyst is a single molecule of B_3_O_3_(OH)_4_^−^; the corresponding rate constants are denoted *k*_OHb(BB)_ and *k*_OHb(B3)_ respectively. The best fits to eqn (4) and (5) are shown as red and blue solid curves respectively in Fig. 4 and 5. Also shown is a dotted line that is the best fit to either eqn (4) or (5) when *k*_OHb(BB)_ or *k*_OHb(B3)_ are set at zero. Eqn (5), *i.e.* involving B_3_O_3_(OH)_4_^−^ as a catalytic species, gives marginally the better fit, with a reduced chi^2^ of 0.0143 compared to that of 0.0183 for eqn (4); the fit without the second catalytic term in eqn (4) and (5) gives a reduced chi^2^ of 0.0217. The corresponding best fit values for the rate constants are shown in Tables 3 and 4 for eqn (4) and (5) respectively.

4 *k*_obsB_ = (*k*_wa(B)_ [AH] [B(OH)_3_] + *k*_wb(B)_ [A^−^] [B(OH)_3_] + *k*_OHb(B)_ [OH] [A^−^] [B(OH)_3_] + *k*_OHb(BB)_ [OH] [A^−^] [B(OH)_3_]^2^)/[P]_t_ + *k*_obs_

5 *k*_obsB_ = (*k*_wa(B)_ [AH] [B(OH)_3_] + *k*_wb(B)_ [A^−^] [B(OH)_3_] + *k*_OHb(B)_ [OH] [A^−^] [B(OH)_3_] + *k*_OHb(B3)_ [OH] [A^−^] [B_3_O_3_(OH)_4_^−^])/[P]_t_ + *k*_obs_

**Table 3:** Rate constants for the boric acid catalysed hydrolysis of PAA and MCPBA, obtained from the best fit to eqn (4) as detailed in the text

Rate	PAA	MCPBA
*k*_wa(B)_/10^−6^ dm^3^ mol^−1^ s^−1^	18.55 ± 4.18	0.11 ± 0.65
*k*_wb(B)_/10^−4^ dm^3^ mol^−1^ s^−1^	5.49 ± 0.43	7.03 ± 0.45
*k*_OHb(B)_/dm^6^ mol^−2^ s^−1^	1.94 ± 0.28	2.26 ± 0.73
*k*_OHb(BB)_/dm^9^ mol^−3^ s^−1^	140 ± 53	112 ± 74

**Table 4:** Rate constants for the boric acid catalysed hydrolysis of PAA and MCPBA, obtained from the best fit to eqn (5) as detailed in the text

Rate	PAA	MCPBA
*k*_wa(B)_/10^−6^ dm^3^ mol^−1^ s^−1^	17.90 ± 3.67	0.10 ± 0.64
*k*_wb(B)_/10^−4^ dm^3^ mol^−1^ s^−1^	6.03 ± 0.24	7.20 ± 0.38
*k*_OHb(B)_/dm^6^ mol^−2^ s^−1^	1.61 ± 0.27	2.11 ± 0.70
*k*_OHb(B3)_/dm^6^ mol^−2^ s^−1^	23.5 ± 5.6	22.8 ±13.85

**Fig. 7:**
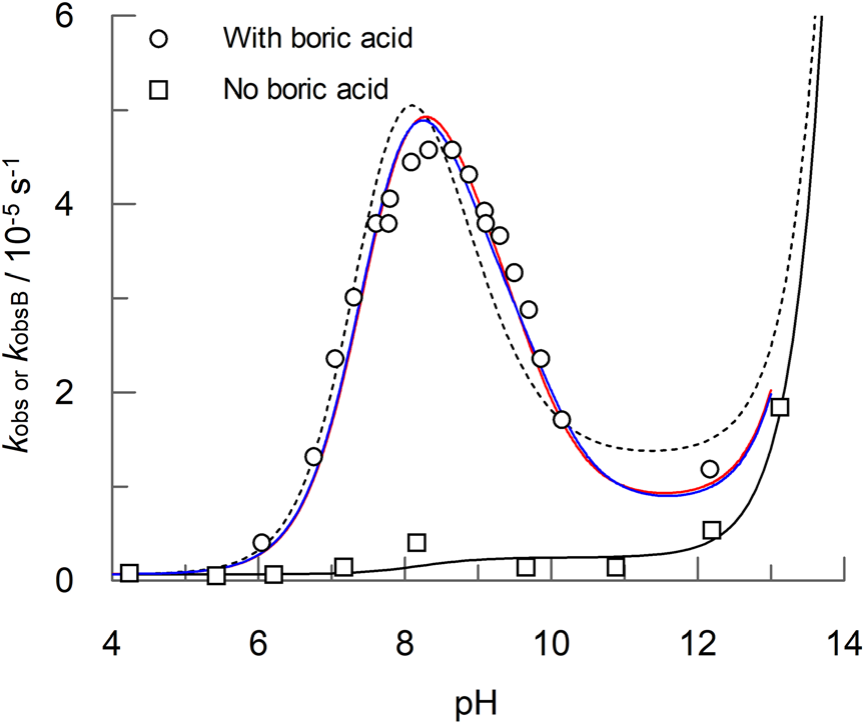
pH dependence of MCPBA hydrolysis in the presence (*k*_obsB_, open circles) and absence (*k*_obs_, filled circles) of 0.1 M boric acid at 25 °C. The initial PAA concentration, [P]_t_, was 3.52 mM. Ionic strength was maintained at 0.2 M with sodium nitrate. All solutions contained 20 μM each of ETDA and EDTMP. The solid black curve is the best fit to eqn (1) for the reaction carried out in the absence of boric acid. The red and blue solid curves are the best fits to eqn (4) and (5) respectively for reactions carried out in the presence of boric acid, with the rate constants listed in Tables 3 and 4. The dotted curve is the best fit to either eqn (4) or (5) when *k*_OHb(BB)_ or *k*_OHb(B3)_, respectively, are set at zero. The corresponding data is included in Table S4 in the ESI.†

As an additional evaluation of the plausibility of either eqn (4) or (5) in accounting for the observed catalysis of peroxyacid hydrolysis, the analysis was repeated for the pH dependence of MCPBA hydrolysis in the presence of boric acid, as shown in Fig. 7 (there was no corresponding concentration data for MCPBA). The solid red and blue curves are the best fits to eqn (4) and (5) respectively with the rate constants shown in Tables 3 and 4 respectively. The best fits describe the pH dependence data well, though, as with PAA, there is a discrepancy at the peak. In contrast to PAA, the catalysis term for the reaction of H_2_O with the conjugate acid of MCPBA, *k*_wa(B),_ was found not to be significant irrespective of whether eqn (4) or (5) was used in the fitting.

**Fig. 8:**
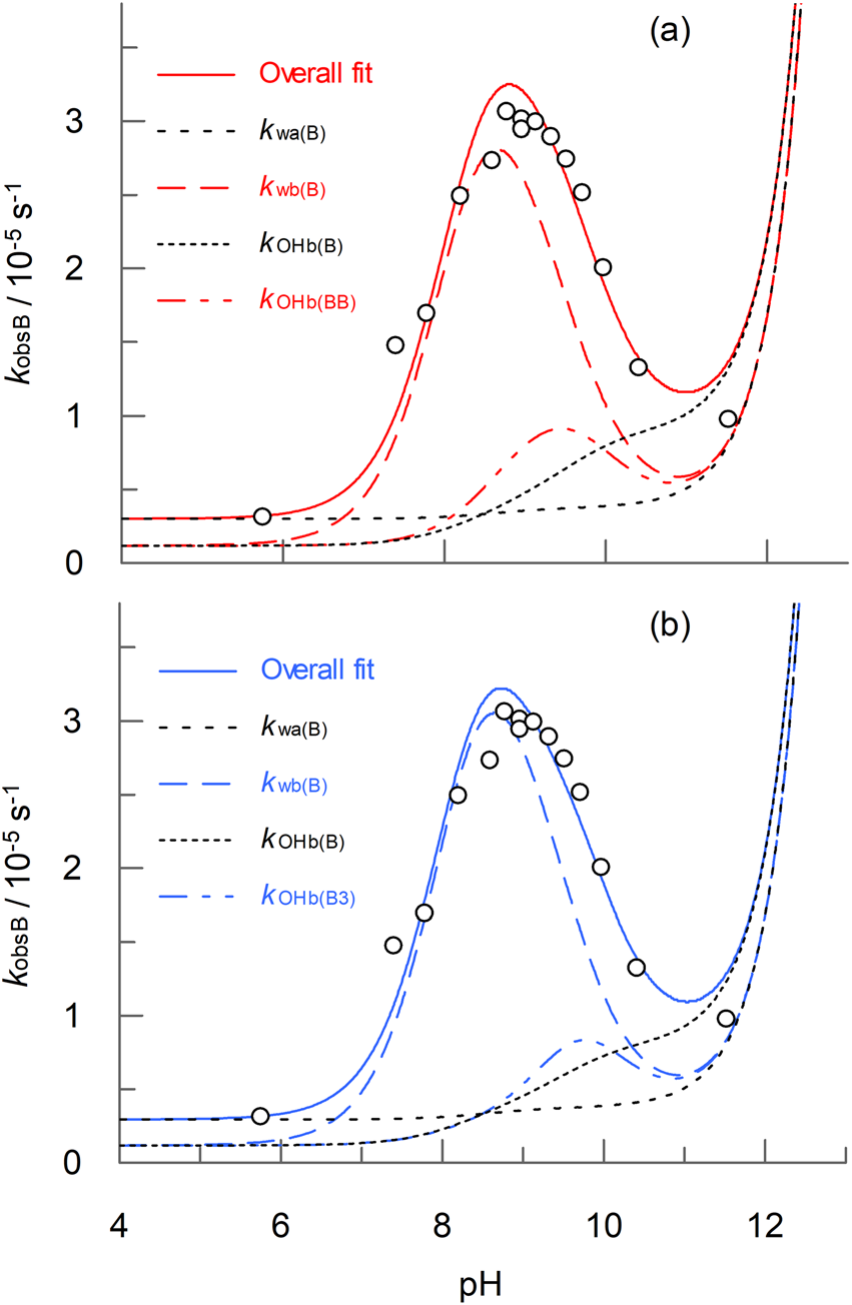
pH dependence of *k*_obsB_ for PAA in the presence of 0.1 M boric acid at 25 °C. Panels (a) and (b) show the contributions from the terms in eqn (4) and (5) respectively.

Before examining possible mechanisms of boric acid catalysis of peroxyacid hydrolysis, it is useful to look at the relative contributions of the individual terms in eqn (4) and (5) to the overall catalysis of PAA, as shown in Fig. 8(a) and (b). For both equations, the largest contribution is from *k*_wb(B)_*i.e.*, the reaction of H_2_O with the conjugate base of PAA, catalysed by a single boric acid molecule (though, because there is kinetic equivalence, this could also correspond to the reaction of OH^−^ with the conjugate acid of PAA). The reaction of the conjugate base of PAA with OH^−^, catalysed by a molecule of B(OH)_3_ (*k*_OHb(B)_) and also by either two molecules of B(OH)_3_ (*k*_OHb(BB)_, Fig. 8(a)) or by B_3_O_3_(OH)_4_^−^ (*k*_OHb(B3)_, Fig. 8(b)) make significant contributions at higher pHs.

### Possible mechanisms of catalysis of peroxyacid hydrolysis by boric acid

With respect to the reaction pathways shown in Scheme 1, the breakdown of the tetrahedral intermediate formed from the reaction of either H_2_O or OH^−^ with either the conjugate acid or the conjugate base of the peroxyacid requires the leaving of the peroxide group, either as HOOH or HOO^−^. The p*K*_a_ of hydrogen peroxide is 11.6, thus making it a relatively poor leaving group. Leaving group basicities have a significant influence on the rate of reaction of analogous reactions such as the reaction of esters with oxygen nucleophiles.^47^ Catalytic mechanisms of boric acid may, therefore, involve triganol B(OH)_3_ acting as a Lewis acid to associate with the peroxide leaving group, lowering the leaving group basicity, and thus facilitating the breakdown of the tetrahedral intermediate to products. B(OH)_3_ is known to have a high affinity for hydrogen peroxide, with monoperoxoborate readily forming above pH 6, and becoming the predominant peroxide species at pHs between 8 and 12 when borate is in excess.^2,3^

**Scheme 2:**
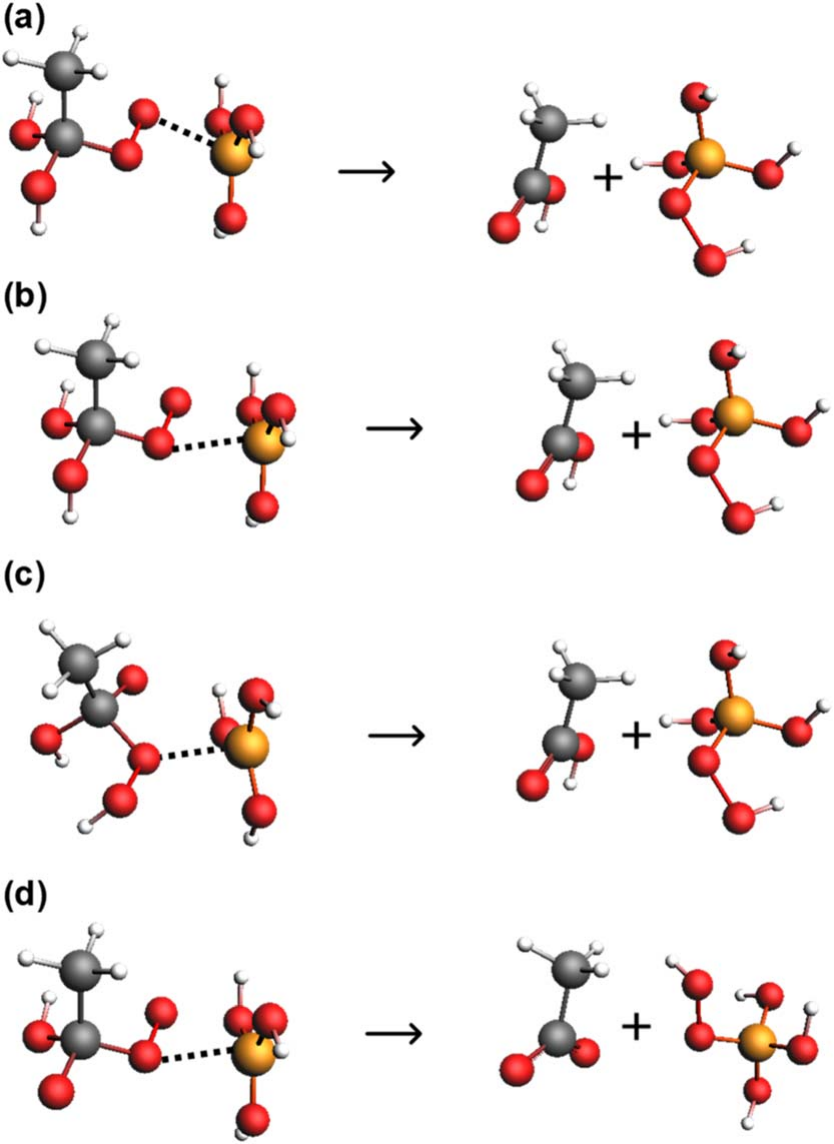
Possible mechanisms for the B(OH)_3_-facilitated breakdown of the tetrahedral intermediate formed by either the attack of H_2_O on the conjugate base of PAA, (a) and (b), or the attack of OH^−^ on either the conjugate acid of PAA, (c) or on the conjugate base (d). The reaction products are acetic acid (or acetate) and monoperoxoborate.

From Fig. 8 it was seen that the largest contribution to the catalysis of PAA hydrolysis, irrespective of whether eqn (4) or (5) was used, involves a single molecule of B(OH)_3_ catalysing the reaction of H_2_O with the conjugate base of PAA (*k*_wb(B)_). The mechanism could either involve the complexation of B(OH)_3_ with the outer peroxo oxygen of the tetrahedral intermediate in an analogous way to which it complexes with hydrogen peroxide, as shown in Scheme 2(a), or complexation with the peroxide oxygen adjacent to the carbonyl carbon, as in Scheme 2(b). An additional proton transfer is required in both cases to form monoperoxoborate, most likely through an intramolecular transfer. In both cases, proton transfer to the peroxide group may already have occurred in the tetrahedral intermediate, either directly from the attacking H_2_O, or from one of the OH^−^ groups, as in Scheme 2(c). The latter scheme could equally apply for the reaction of OH^−^ with the conjugate acid of PAA, that is kinetically equivalent to the attack of water on the conjugate base. For the catalysis of the reaction of OH^−^ with the conjugate base of PAA by B(OH)_3_, *k*_OHb(B),_ complexation could take place at the peroxide oxygen adjacent to the carbonyl carbon, as in Scheme 2(d), though this would require an additional proton transfer step to form monoperoxoborate.

**Scheme 3:**
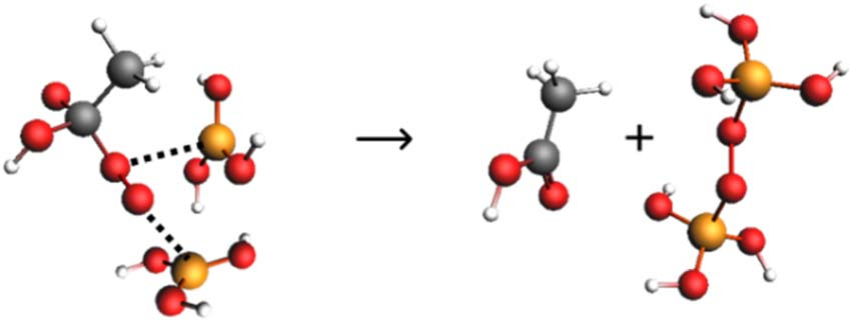
Possible mechanism for the catalysis of PAA hydrolysis by two molecules of B(OH)_3_.

For the reaction of the conjugate base of PAA with OH^−^, catalysed by two molecules of boric acid, *i.e. k*_OHb(BB)_ in eqn (4), the mechanism shown in Scheme 3 would facilitate the leaving of the peroxide group as monoperoxodiborate, which is known from the literature.^48^ It is possible that there may be a pre-association of one of the boric acids with the outer peroxo oxygen of the peracetic acid anion prior to the transition state, thus avoiding the requirement for three molecules in the transition state (McKillop and Sanderson have proposed a similar structure, though for non-aqueous conditions^49^).

For the alternative scenario described by eqn (5), involving the reaction of the conjugate base of PAA with OH^−^, catalysed by B_3_O_3_(OH)_4_^−^, analogous mechanisms to those shown in Scheme 2 could apply, with one of the two trigonal borons acting as a Lewis acid.

As for which of eqn (4) or (5), is preferred, it could be argued that the negative charge on B_3_O_3_(OH)_4_^−^ when catalysing the decomposition of a negatively charged tetrahedral intermediate would make this more unlikely compared to the neutral B(OH)_3_. On the other hand, the requirement in eqn (4) of the involvement of three molecules in the transition state for B(OH)_3_ catalysis might mean that B_3_O_3_(OH)_4_^−^ is favoured (unless there was a pre-complexation of one of the B(OH)_3_ molecules). Additionally, Lopalco *et al.* have suggested that polyborates are stronger acids than boric acid^26^ and so B_3_O_3_(OH)_4_^−^ may be more effective in complexing with the peroxide group.

Finally, there is conflicting evidence on whether boric acid catalyses the reaction between H_2_O and the conjugate acid of the peroxyacids, *k*_wa(B)_. The term was significant for PAA but not for MCPBA. A catalytic mechanism could again involve complexation with the peroxide oxygen adjacent to the carbonyl carbon, though an additional proton transfer from one of the transition state OH groups to solvent would be necessary.

### Practical implications

From the rate data shown in Fig. 2 (and Table S1†), peroxyacid hydrolysis in the absence of boric acid proceeds very slowly, with a half-life of 75 hours for PAA at pH 9.06 at 25 °C, though this falls to 11.1 hours at pH 12.05. Yuan *et al.*^13^ have reported higher hydrolysis rates, for example 67 minutes to convert 95% of peracetic acid to hydrogen peroxide at 60 °C at pH 11, though the rates in the present study are consistent with the work of Secco *et al.*^12^ In the presence of 0.1 M boric acid at 9.06 (the optimum for boric acid catalysis), the half-life decreases to *ca.* 6.5 hours (Fig. 4 and Table S2†), and further still to 4.4 hours at a boric acid concentration of 0.2 M (Fig. 5 and Table S3†). Washing liquors that depend on sodium perborate and tetracetylethylenediamine (TAED) to generate PAA will typically contain 0.025 M boric acid,^5^ at which the half-life for borate catalysed hydrolysis will be *ca.* 15 hours at pH 9.06 and 25 °C. Therefore, even at the slightly higher temperature of a modern wash cycle (30–40 °C), the loss of PAA due to borate catalysed hydrolysis is likely to be relatively small. Higher borate concentrations have been reported for some textile bleaching systems that use PAA, for example 0.1 M borate used as a stabiliser in jute bleaching (1 hour, 45 °C, neutral pH),^6^ though, again PAA loss will be small, especially at the neutral pH used. More-alkaline conditions will result in faster PAA hydrolysis rates; however, at pHs above 10, the predominant boron species will be B(OH)_4_^−^, which does not participate in the catalysis. These conclusions will also be relevant to bleaching systems that use alternatives to PAA, such as the long hydrophobic chain analogues, peroxynonanoic, peroxydecanoic acid and peroxydodecanoic acid.^50^

The speciation modelling in the current study (Fig. 3) shows that temperature does affect the composition of boron species in solution, with a *ca.* 40% reduction in B_3_O_3_(OH)_4_^−^ at pH 9.07 for a temperature increase from 25 °C to 50 °C (and a 67% reduction between 25 °C and 75 °C). However, the current work has shown that the main catalytic species for PAA hydrolysis is B(OH)_3_, with a small contribution from either 2B(OH)_3_ or from B_3_O_3_(OH)_4_^−^. Therefore, temperature is unlikely to significantly change the concentration of the main catalytic species, even if B_3_O_3_(OH)_4_^−^ is involved.

## Conclusions

It is important to understand the mechanisms that lead to the loss of peroxyacids through hydrolysis because they are widely used oxidants in a range of applications, such as domestic laundry washing, textile bleaching, wood pulp bleaching and chlorine free disinfection processes.

In this work, boric acid has been shown to catalyse the hydrolysis of peroxyacids, showing a maximum catalytic effect at pH 9 for PAA and pH 8.4 for MCPBA, with an approximately 12-fold rate enhancement in both cases at a boric acid concentration of 0.1 M.

The elucidation of the mechanisms of boric acid catalysis of peroxyacid hydrolysis is complex because there are four possible hydrolysis pathways, and several potential boron species that could participate in the catalysis. Aqueous chemistry speciation modelling using OLI studio showed that in addition to B(OH)_3_ and B(OH)_4_^−^, the following polyborate species will be present at the pH where the maximum catalytic effect is observed: B_2_O(OH)_5_^−^, B_3_O_3_(OH)_4_^−^ and B_4_O_5_(OH)_4_^2−^. Of these, B_3_O_3_(OH)_4_^−^, is predicted to be the dominant polyborate species, representing a maximum of 5% of the total boron species at 25 °C.

Taking into account the boric acid speciation, curve-fitting analysis carried out simultaneously on both the pH and concentration dependence data indicated that the main catalytic pathway involves one B(OH)_3_ molecule facilitating the reaction of either H_2_O with the conjugate base of the peroxyacid or of OH^−^ with the conjugate acid (these pathways are kinetically indistinguishable). It is proposed that the boric acid molecule acts as a Lewis acid catalyst, lowering the p*K*_a_ of the peroxide leaving group. The products of the catalysed reaction will be acetic acid and monoperoxoborate, which is a well characterized and stable peroxoborate species. The analysis also revealed that a second significant catalytic pathway involves either (a) two molecules of B(OH)_3_, or (b) a single B_3_O_3_(OH)_4_^−^ molecule facilitating the leaving of the peroxide group of the tetrahedral intermediate formed from the reaction of OH^−^ with the conjugate base of the peroxyacid. The best fit to the data was poorer without a term for either B(OH)_3_ or B_3_O_3_(OH)_4_^−^.

The effect of temperature on the reaction was not directly studied; however, speciation modelling shows that when the temperature is increased from 25 °C to 75 °C, the concentration of one of the potential catalytic species, B_3_O_3_(OH)_4_^−^, will fall by half, whilst the concentration of B_2_O(OH)_5_^−^ will increase. A temperature dependence study would, therefore, have to consider the changing concentrations of catalytic species. Nevertheless, a Δ*S*^‡^ value would be of help in differentiating between the two proposed catalytic mechanisms for the reaction of OH^−^ with the conjugate base of the peroxyacid.

The predicted effect of boric acid on hydrolytic loss of peroxyacids in various practical applications is small, with the half-life of PAA in typical TAED/perborate washing liquors being several hours for low temperature washing conditions. Nevertheless, the current work provides a basis for predicting peroxyacid loss due the hydrolysis for any application where boric acid is also present. The work also extends our understanding of the catalytic mechanisms of boric acid in aqueous peroxide systems.

Finally, it is acknowledged that this is a complex system, particularly with respect to boric acid speciation, and that other combinations of pathways involving borate species might explain the observed pH and boric acid concentration dependencies.

## Conflicts of interest

There are no conflicts to declare.

## Supplementary Material

RA-013-D3RA01046E-s001

## References

[cit1] Davies D. M., Deary M. E., Quill K., Smith R. A. (2005). Borate-catalyzed reactions of hydrogen peroxide: kinetics and mechanism of the oxidation of organic sulfides by peroxoborates. Chem.–Eur. J..

[cit2] Durrant M. C., Davies D. M., Deary M. E. (2011). Dioxaborirane: a highly reactive peroxide that is the likely intermediate in borate catalysed electrophilic reactions of hydrogen peroxide in alkaline aqueous solution. Org. Biomol. Chem..

[cit3] Deary M. E., Durrant M. C., Davies D. M. (2013). A kinetic and theoretical study of the borate catalysed reactions of hydrogen peroxide: the role of dioxaborirane as the catalytic intermediate for a wide range of substrates. Org. Biomol. Chem..

[cit4] Davies D. M., Deary M. E. (1991). Kinetics of the hydrolysis and perhydrolysis of tetraacetylethylenediamine, a peroxide bleach activator. J. Chem. Soc., Perkin Trans. 2.

[cit5] Hickman W. (2002). Peracetic acid and its use in fibre bleaching. Rev. Prog. Color. Relat. Top..

[cit6] Cai Y., David S. (1997). Low temperature bleaching of jute fabric using a peracetic acid system. Text. Res. J..

[cit7] Kang G. J., Malekian A., Ni Y. (2004). Formation of peracetic acid from hydrogen peroxide and pentaacetyl glucose to activate oxygen delignification. Tappi J..

[cit8] Luukkonen T., Pehkonen S. O. (2017). Peracids in water treatment: a critical review. Crit. Rev. Environ. Sci. Technol..

[cit9] Yuan Z., Ni Y., Van Heiningen A. (1997). Kinetics of peracetic acid decomposition: part I: spontaneous decomposition at typical pulp bleaching conditions. Can. J. Chem. Eng..

[cit10] Kowalska M., Ramos E. (2014). Optimization of TCF single-stage bleaching of abaca soda pulp with a mixture of peracetic acid and sodium perborate. Cellul. Chem. Technol..

[cit11] Secco F., Venturini M., Celsi S. (1972). Kinetics and salt effects on alkaline-hydrolysis of perbenzoate ion. J. Chem. Soc., Perkin Trans. 2.

[cit12] Secco F., Venturini M., Celsi S. (1973). Kinetics and mechanism of acid and alkaline hydrolyses of some perbenzoic acids. J. Chem. Soc., Perk Trans. 2.

[cit13] Yuan Z., Ni Y., Van Heiningen A. (1997). Kinetics of the peracetic acid decomposition: part II: pH effect and alkaline hydrolysis. Can. J. Chem. Eng..

[cit14] Secco F., Celsi S. (1971). Kinetics of hydrolysis of peroxybenzoic acid in aqueous acidic solution. J. Chem. Soc. B.

[cit15] Zhao X., Zhang T., Zhou Y., Liu D. (2007). Preparation of peracetic acid from hydrogen peroxide: part I: kinetics for peracetic acid synthesis and hydrolysis. J. Mol. Catal. A: Chem..

[cit16] da Silva W. P., Carlos T. D., Cavallini G. S., Pereira D. H. (2020). Peracetic acid: structural elucidation for applications in wastewater treatment. Water Res..

[cit17] Unis M. M. , Peroxide Reactions of Environmental Relevance in Aqueous Solution, Northumbria University, Newcastle upon Tyne, 2010

[cit18] Ball R. E., Edwards J. O., Haggett M. L., Jones P. (1967). A kinetic and isotopic study of the decomposition of monoperoxyphthalic acid. J. Am. Chem. Soc..

[cit19] Saunders K. G., Stadnicki R. T., Mcisaac J. E. (1975). Base-catalyzed decomposition of p-nitroperoxobenzoic acid. J. Inorg. Nucl. Chem..

[cit20] E K., Haggett M. L., Battaglia C. J., Khairat M. I.-R., Pyun H. Y., Edwards J. O. (1963). Kinetics and mechanism of the spontaneous decompositions of some peroxoacids, hydrogen peroxide and t-butyl hydroperoxide. J. Am. Chem. Soc..

[cit21] Evans D. F., Upton M. W. (1985). Studies on singlet oxygen in aqueous-solution 3.
The decomposition of peroxy-acids. J. Chem. Soc., Dalton Trans..

[cit22] Goodman J. F., Robson P., Wilson E. R. (1962). Decomposition of aromatic peroxyacids in aqueous alkali. Trans. Faraday Soc..

[cit23] Akiba K., Simamura O. (1970). Decomposition of sodium peroxybenzoate in sodium hydroxide solution. Tetrahedron.

[cit24] Ball D. L., Edwards J. O. (1956). The kinetics and mechanism of the decomposition of Caro's acid. I. J. Am. Chem. Soc..

[cit25] Farmer J. B. (1982). Metal borates. Adv. Inorg. Chem. Radiochem..

[cit26] Lopalco A., Lopedota A. A., Laquintana V., Denora N., Stella V. J. (2020). Boric acid, a Lewis acid with unique and unusual properties: formulation implications. J. Pharm. Sci..

[cit27] Applegarth L. M., Pye C. C., Cox J. S., Tremaine P. R. (2017). Raman spectroscopic and *ab initio* investigation of aqueous boric acid, borate, and polyborate speciation from 25 to 80 °C. Ind. Eng. Chem. Res..

[cit28] Zhou Y. Q., Fang C. H., Fang Y., Zhu F. Y. (2011). Polyborates in aqueous borate solution: a Raman and DFT theory investigation. Spectrochim. Acta, Part A.

[cit29] Chen L., Li D., Guo Y., Deng T., Meng L. (2019). Experimental data and thermodynamic model in the salt–water ternary system (NaBO_2_+ Na_2_B_4_O_7_+ H_2_O) at t= 298.15 K and p= 0.1 mPa. J. Chem. Eng. Data.

[cit30] Mesmer R., Baes Jr C., Sweeton F. (1972). Acidity measurements at elevated temperatures. VI. Boric acid equilibriums. Inorg. Chem..

[cit31] Ingri N. (1963). Equilibrium studies of polyions 11. Acta Chem. Scand..

[cit32] Graff A., Barrez E., Baranek P., Bachet M., Bénézeth P. (2017). Complexation of nickel ions by boric acid or (poly) borates. J. Solution Chem..

[cit33] Wang P., Kosinski J. J., Lencka M. M., Anderko A., Springer R. D. (2013). Thermodynamic modeling of boric acid and selected metal borate systems. Pure Appl. Chem..

[cit34] Li D., Zhou G., Gu S., Zhang T., Meng L., Guo Y., Deng T. (2020). Thermodynamic and dynamic modeling of the boron species in aqueous potassium borate solution. ACS Omega.

[cit35] Spessard J. E. (1970). Investigations of borate equilbria in neutral salt solutions. J. Inorg. Nucl. Chem..

[cit36] Ingri N. (1962). Equilibrium studies of polyanions. 8. On the first equilibrium steps in the hydrolysis of boric acid, a comparison between equilibria in 0.1 M and 3.0 M NaClO_4_. Acta Chem. Scand..

[cit37] Salentine C. G. (1983). High-field B-11 NMR of alkali borates – aqueous polyborate equilibria. Inorg. Chem..

[cit38] Maya L. (1976). Identification of polyborate and fluoropolyborate ions in solution by raman-spectroscopy. Inorg. Chem..

[cit39] Duffin A. M., Schwartz C. P., England A. H., Uejio J. S., Prendergast D., Saykally R. J. (2011). pH-dependent x-ray absorption spectra of aqueous boron oxides. J. Chem. Phys..

[cit40] Palmer D., Bénézeth P., Wesolowski D. (2000). Boric acid hydrolysis: a new look at the available data. Power Plant Chem..

[cit41] Davies D. M., Deary M. E. (1988). Determination of peracids in the presence of a large excess of hydrogen peroxide using a rapid and convenient spectrophotometric method. Analyst.

[cit42] Deary M. E., Amaibi P. M., Dean J. R., Entwistle J. A. (2021). New insights into health risk assessments for inhalational exposure to metal (Loid) s: the application of aqueous chemistry modelling in understanding bioaccessibility from airborne particulate matter. Geosciences.

[cit43] Leatherbarrow R. J. , GraFit Version 7, Erithacus Software Ltd, Horley, UK, 2009

[cit44] Everett A., Minkoff G. (1953). The dissociation constants of some alkyl and acyl hydroperoxides. Trans. Faraday Soc..

[cit45] Davies D. M., Jones P. (1978). Enhanced nucleophilic reactivity (alpha. effect) in the reaction of peroxobenzoate anions with p-nitrophenyl acetate. J. Org. Chem..

[cit46] Durrant M. C. , Personal Communication, 2015

[cit47] Jencks W. P., Gilchrist M. (1968). Nonlinear structure-reactivity correlations. The reactivity of nucleophilic reagents toward esters. J. Am. Chem. Soc..

[cit48] Rey S., Davies D. M. (2006). Photochemistry of peroxoborates: borate inhibition of the photodecomposition of hydrogen peroxide. Chem.–Eur. J..

[cit49] McKillop A., Sanderson W. R. (1995). Sodium perborate and sodium percarbonate: cheap, safe and versatile oxidising agents for organic synthesis. Tetrahedron.

[cit50] Bianchetti G. O., Devlin C. L., Seddon K. R. (2015). Bleaching systems in domestic laundry detergents: a review. RSC Adv..

